# “Induced” Eyes Suggest a Path to Retinal Repair

**DOI:** 10.1371/journal.pbio.1000175

**Published:** 2009-08-18

**Authors:** William Mair

**Affiliations:** Freelance Science Writer, La Jolla, California, United States of America

**Figure pbio-1000175-g001:**
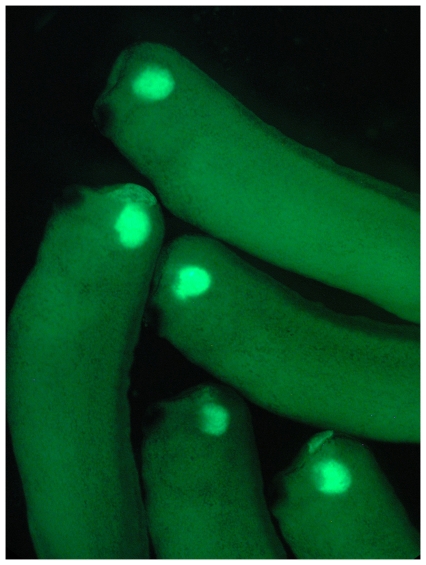
How many genes does it take to make a functioning eye? Apparently, only seven. Frog cells forced to express these seven genes (and a fluorescent protein that glows green) form eyes in tadpoles.

[Fig pbio-1000175-g001]Anti-evolutionists have long cited the eye as evidence against evolution. The organ is so intricate and complex, they argue, that it could not exist but for the careful guidance of a grand designer. Indeed, even Darwin himself labored to explain how such complexity might be the product of natural selection. The presence of primitive eye-like structures in lower species and, ironically, the non-intelligent design of the vertebrate eye itself—it is essentially built backward, as light must first travel past blood vessels and nerve tissue before it hits the photoreceptors—closes the lid on that argument for evolutionary biologists, yet the factors regulating eye formation during development are only now beginning to become clear.

In this issue of *PLoS Biology*, Michael Zuber and colleagues elegantly demonstrate that by turning on just seven genes in frog pluripotent stem cells, tadpoles that would otherwise be blind can develop functional eyes from the transplanted cells. If the same techniques translate to human cells, they may provide a strategy for restoring vision to patients with retinal damage or hereditary eye diseases.

Regenerative medicine aims to replace damaged tissue with new, functioning cells derived from embryonic stem cells, which have the potential to develop into any adult cell type. A challenge for such regenerative approaches, however, is that tissues are composed of several different types of cells, rather than just one. Stimulating stem cells to develop into a particular cell type is challenging enough, but inducing them to develop into functioning tissues requires an understanding of the mechanisms that determine the fate of multiple cell types.

Zuber and colleagues set about tackling this issue for cells in the retina, where photoreceptors (rods and cones) detect incoming light and convert it into electrical signals. Once converted, these signals travel down the optic nerve to the brain's visual cortex, which interprets these signals as visual images. Patients with retinal dystrophy have degenerative retinas, where the photoreceptors are either absent or non-functioning.

The retina contains seven major classes of cells (including the rods and cones), which are derived during development from “retinal precursor cells” found in an area of the embryo known as the eye field. The team aimed to convert stem cells into retinal precursor cells in the hope that these could then be used to generate functioning eye tissue. During development, cells in the eye field switch on seven specific transcription factors, proteins that bind to DNA and turn on a panel of genes involved in building the eye. Because these eye field transcription factors (EFTFs) are required for proper eye formation, the scientists artificially expressed the EFTFs in stem cells in an effort to turn them into retinal precursor cells.

First, they wanted to see if turning on EFTFs in stem cells activated the same genes the transcription factors normally targeted in the eye field cells. Whole genome microarray analysis showed that the panel of genes activated in the EFTF stem cells was indeed similar to those active in eye field cells. And, when the researchers transplanted EFTF stem cells into the flank of developing embryos, eye-like structures developed instead of skin. These structures could not be tested for function, however, because they were in the wrong location to connect to the correct neurons.

Next, the scientists removed the native eye field cells in frog embryos and replaced them with EFTF stem cells. This treatment produced functional eye tissue; these “induced” eye structures not only contained all seven cell types found in normal retinas but also generated optic nerves that connected to the brain. Electroretinogram analysis, which detects electrical signals transmitted from the retina when exposed to light, suggested that the induced eyes were functioning just as well as normal eyes. But the real proof came from testing the behavior of tadpoles with and without induced eyes.

Tadpoles are surprisingly choosy. When they are put in a tank with one white side and one black side, they swim toward the light half of the tank. This behavior is vision-dependent, because blind tadpoles show no side preference and swim randomly about the tank. Proving that the stem cell–derived induced eyes were not only morphologically but functionally normal, tadpoles with induced eyes behaved the same as normally sighted ones and swam toward the light side.

Zuber and colleagues have not only managed to drive stem cells to develop into a particular cell type, they have also managed to generate a complete and functioning tissue. Their next challenge is to determine how to maintain cultures of stem cell–derived retinal precursor cells that could then be readily used to regenerate defective eye tissue. Understanding precisely which molecular cues in eyes turn stem cells into all the cell types of the retina is the first step to shedding light on a cure for degenerative eye diseases.

**Viczian AS, Solessio EC, Lyou Y, Zuber ME (2009) Generation of Functional Eyes from Pluripotent Cells. doi:10.1371/journal.pbio.1000174**

